# Surface and fracture properties of lithium disilicate and resin-Matrix CAD/CAM ceramics

**DOI:** 10.1007/s44445-026-00145-z

**Published:** 2026-04-25

**Authors:** Ayat G. Montaser, Sara Tamimi, Shimaa A. El Saeed, Ahmed D. Abogabal, Ahmed Y. Allam, Suad M. Hassan, Sally N. El Adawy, Amany H. Elzoka

**Affiliations:** 1 Crowns and Bridges Department, Faculty of Dental Medicine for Girls, Al Azhar Universit, Cairo, Egypt; 2 Dental Biomaterials Department, Faculty of Dental Medicine for Girls, Al Azhar Universit, Cairo, Egypt; 3 Department of Biomaterials, Faculty of Dental Medicine (Boys), Al-Azhar University, Assiut, Egypt; 4 Crowns and Bridges Department, Faculty of Dental Medicine (Boys), Assiut, Al-Azhar University, Egypt., Assiut, Egypt; 5 Department of Dental Basic Science, Dental Biomaterials, Faculty of Dentistry, Petra University, Amman, Jordan

**Keywords:** IPS e.max CAD, MAZIC Duro, Material thicknesses, Hardness, Fracture toughness

## Abstract

**Purpose:**

To characterize hardness and fracture toughness of ceramic CAD/CAM materials with different composition (IPS e.max CAD and MAZIC Duro) and thicknesses (1mm and 1.5mm).

**Materials and methods:**

Blocks of CAD/CAM esthetic restorative materials (IPS e. max CAD "EC" and MAZIC Duro "MD"), forty discs were fabricated with dimensions of 10 mm × 1 mm and 10 mm × 1.5 mm. All discs were prepared using a precision cutting machine. Discs were then subjected to hardness, fracture toughness tests.

**Results:**

Data were analyzed using independent t-tests and Two-way ANOVA (p < 0.05). For hardness test, at 1 mm and 1.5mm thicknesses, MAZIC Duro (MD) recorded a not statistically significant higher mean value of hardness (459.836 ± 20.549 HV) (468.818 ± 17.574 HV) compared to IPS e.max CAD (EC) (456.596 ± 38.102 HV) (463.346 ± 11.399 HV) (p = 0.816 and p = 0.420, respectively). For fracture toughness test, at 1 mm thickness, MAZIC Duro (MD) recorded a highly statistically significant higher mean value of fracture toughness (5.360 ± 0.133 MPa) compared to IPS e.max CAD (EC) (4.744 ± 0.151 MPa) (p < 0.001). While at 1.5 mm thickness, MAZIC Duro (MD) recorded a not statistically significant higher mean value of fracture toughness (5.291 ± .089 MPa) compared to IPS e.max CAD (EC) (5.285 ± 0.330 MPa) (p = 0.956).

**Conclusion:**

MAZIC Duro (MD) showed slightly higher hardness than IPS e.max CAD (EC) at both 1 mm and 1.5 mm thicknesses, but the difference was not statistically significant. For fracture toughness, MD was highly significant at 1 mm thickness, while at 1.5 mm, both materials performed similarly with no significant difference.

**Supplementary Information:**

The online version contains supplementary material available at https://doi.org/10.1007/s44445-026-00145-z.

## Introduction

Advancements in dental restorative materials have profoundly impacted clinical practice, particularly with the integration technology of CAD/CAM. This technology enables the precise fabrication of ceramic restorations with enhanced esthetics and mechanical performance. Among the most widely used materials in this domain are IPS e.max CAD, a lithium disilicate glass ceramic, and MAZIC Duro, a resin-matrix ceramic. Each material offers distinct advantages in terms of strength, wear resistance, surface hardness, and adaptability to various clinical scenarios (Asaad R et al. 2021).

Surface hardness is a critical property for restorative materials, as it reflects their resistance to permanent indentation, scratching, or penetration. It correlates with mechanical strength and influences abrasiveness against opposing dentition. Studies indicate that ceramics with high hardness values can withstand significant masticatory forces (Asaad R et al. 2021; Al-Johani H et al. 2024).

Ceramics are inherently brittle and sensitive to surface flaws caused by thermal, chemical, or mechanical factors. These flaws can lead to localized stress concentrations, crack propagation, and eventual failure. Fracture toughness, the ability to resist crack propagation, is a key determinant of intraoral performance. Materials with higher fracture toughness generally offer better long-term clinical serviceability (Rexhepi et al. 2023; Fouda et al. 2024).

IPS e.max CAD exhibits moderate fracture toughness and high hardness; this makes it appropriate for use in both posterior and anterior restoration. However, reports suggest lower flexural strength and microhardness compared to newer materials such as Tessera and MAZIC Claro (Zulkiffli S et al. 2024). MAZIC Duro is, composed of approximately 80% ceramic fillers and 20% resin matrix, provides elasticity, strength, stain resistance, and esthetics. It is indicated for single-tooth restorations and does not require sintering or glazing. (Çağlayan E et al. (2025), Yin R et al. (2019)) reported that MAZIC Duro demonstrated higher biaxial flexural strength and less abrasion on opposing teeth compared to polymer-infiltrated ceramics.

A previous systematic review highlights the broad use of CAD/CAM materials in clinical practice has expanded owing to their improved mechanical properties. Lithium disilicate ceramics offer a balance between esthetics and strength, while hybrid ceramics provide superior machinability and wear resistance (Avram L et al. 2021).

Restoration thickness significantly influences ceramic performance. Increasing thickness generally enhances fracture toughness, especially in brittle materials like glass ceramics. Hybrid ceramics may maintain adequate toughness even at reduced thicknesses, supporting minimally invasive preparations.

The expiration of IPS e.max patents led to the emergence of alternative CAD/CAM materials such as CEREC Tessera™, MAZIC Claro CAD, and MAZIC Duro CAD. Manufacturers claim comparable mechanical properties to IPS e.max; however, scientific evidence remains limited. Especially, there is a lack of direct comparative studies in the literature evaluating the mechanical performance of the lithium disilicate-based IPS e.max CAD versus the resin-matrix ceramic MAZIC Duro, particularly concerning the influence of restoration thickness on their hardness and fracture toughness. This study aims to characterize and compare the hardness and fracture toughness of IPS e.max CAD and MAZIC Duro at two thicknesses (1 mm and 1.5 mm). The null hypothesis states that no significant difference exists between the microhardness and fracture toughness of IPS e.max CAD and MAZIC Duro and that these properties are not significantly influenced by the restoration thickness.

## Materials and methods

### Sample size

With an effect size of 0.55 (based on the fracture toughness results as the primary outcome), a power of 0.83, and an alpha of 0.05, the appropriate sample size was chosen. At least 40 overall samples were calculated (10 in each category).

### Samples’ grouping

Based on the type of ceramics used, forty ceramic discs were fabricated following the manufacturers’ recommendations, representing two distinct groups, each consisting of twenty samples:

• Group (EC)

IPS e.max CAD glass ceramic.

• Group (MD)

MAZIC Duro resin-matrix ceramic.

Based on the various thicknesses utilized for disc fabrication, each group was further split into two subgroups, each consisting of ten samples:

• Subgroup (1 mm).

Discs with 1 mm thickness.

• Subgroup (1.5 mm).

Discs with 1.5 mm thickness.

### Samples’ preparation

**Fig. 1:**
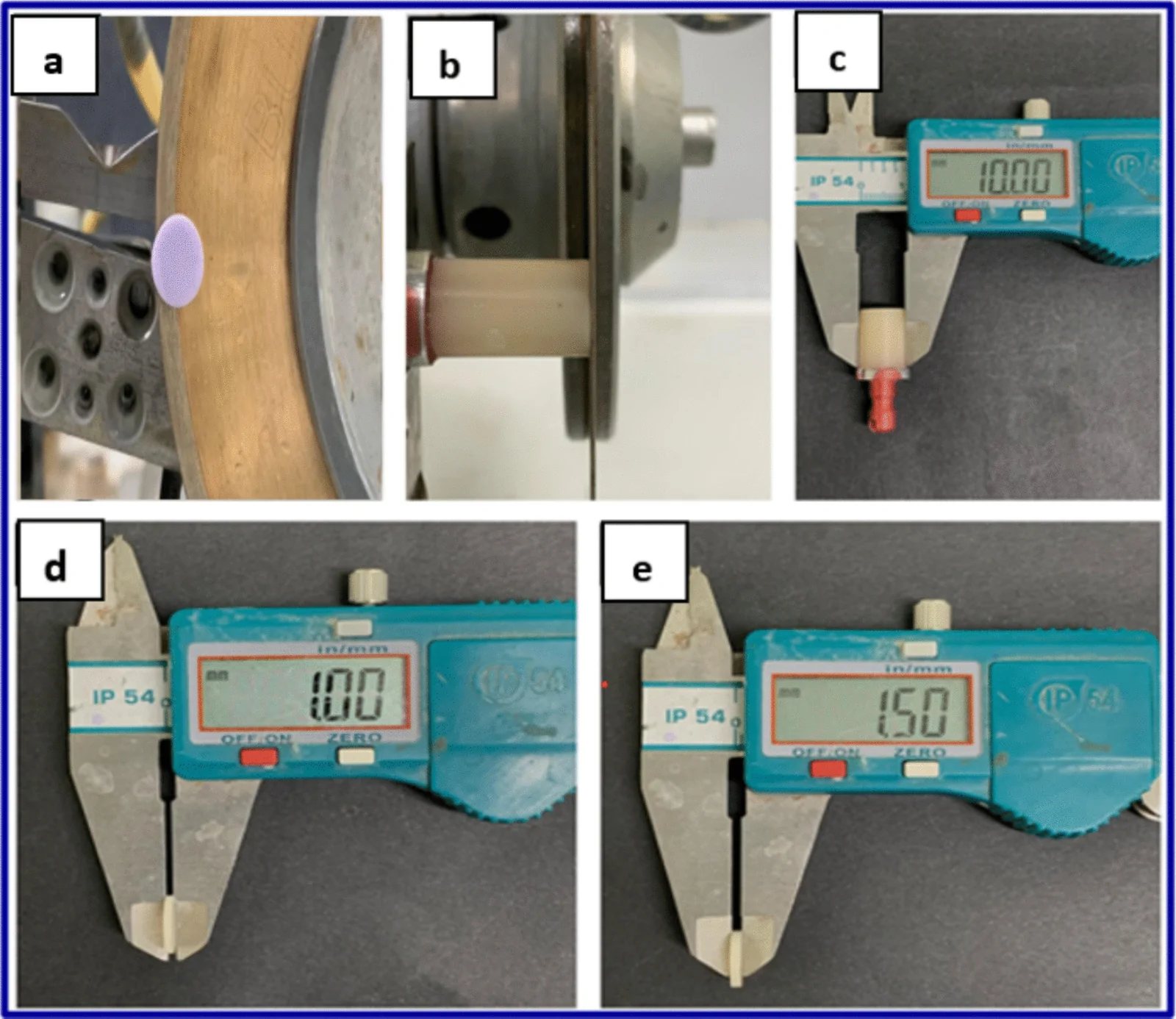
Samples’ preparation; (**a**) Sectioning IPS e. max CAD block into disks. (**b**) Sectioning MAZIC Duro block into disks. (**c**) Digital caliper showing 10 mm diameter. (**d**, **e**) Digital caliper showing 1 mm and 1.5mm thicknesses, respectively

Two types of block of CAD/CAM (IPS e.max CAD and MAZIC Duro) were sectioned into circular samples of 10 mm diameter and two thicknesses (1 and 1.5mm). The sectioning process was performed using a precision cutting machine (Isomet 5000, Buehler, Lake Bluff, IL, USA) under continuous cooling with an adequate water supply. Figure 1 (a-c).

Following this, IPS e.max CAD specimens were subjected to a crystallization cycle at 850 °C for 10 min in a Programat® EP 5000 furnace (Ivoclar Vivadent), in accordance with the manufacturer’s guidelines. In contrast, MAZIC Duro specimens did not require any sintering, crystallization, or additional post-processing following sectioning, as the material is supplied in a fully polymerized and ready-to-use state.

After that, a uniform finishing and polishing procedure was applied to each sample in both groups. Initially, each sample surface was finished by using a diamond grit finishing stone (Mani, Inc., Japan) in two unidirectional passes at low rotating speed and low pressure.

Each sample in both groups was then polished using a KENDA polishing kit (Vaduz, Lichenstien) mounted in a low-speed handpiece without polishing paste, which is specifically indicated for both ceramic and composite restorative materials. following the manufacturer’s guidelines.

To ensure consistency, the same operator polished each sample for 30 s in a single direction. Each sample was completely cleaned in an ultrasonic device (MCS digital ultrasonic unit, Italy) and rinsed under running water before being polished and finished and allowed to air dry. Their measurements were then reevaluated using a digital caliber (TOTAL Tools, Shanghai, China) to make sure that these procedures did not alter the size of any samples. Figure 1(d,e).

### Testing procedures

#### Surface micro-hardness assessment

Surface microhardness was assessed using a Digital Display Vickers Micro-hardness Tester (Model HVS-50, Laizhou Huayin Testing Instrument, China) equipped with a Vickers diamond indenter and a 20 × objective lens. Each sample was subjected to a 500 g load for 20 s (Abd-Elfattah HY et al. 2026). Three evenly spaced indentations were made on each specimen’s surface, arranged in a circular pattern with a maximum separation of 0.5 mm. The diagonal lengths of the indentations were measured using the built-in scaled microscope, and Vickers values were converted to microhardness using the specified formula

$$\mathrm{HV}=1.854\text{ P}/{\mathrm{d}}^{2}$$

where **HV** = Vickers hardness in Kgf/mm.^2^

**P** = the load in Kgf.

**d** = the length of the diagonals in mm.

#### Fracture toughness assessment

Fracture toughness was evaluated using the indentation fracture technique. Crack length generated by the indentation (c, mm) and half of the diagonal indentation (a, mm) were recorded to compute the c/a ratio, as derived from the Vickers hardness measurements described earlier. Fracture toughness was then calculated using the following equation.

$${K}_{IC}=0.016{\left(\frac{E}{H}\right)}^{0.5}\left(\frac{P}{{c}^{1.5}}\right)$$

where.

E = the elastic modulus in GPa.

H = the hardness in GPa.

P = the indentation load in N.

c = the crack length in m.

### Statistical analysis

Data analysis was performed using SPSS software (version 20). Numerical variables were summarized as mean, standard deviation, and 95% confidence intervals. Normality was assessed through data distribution evaluation and the Shapiro–Wilk and Kolmogorov–Smirnov tests. Independent t-tests were used for pairwise comparisons between the two materials (IPS e.max CAD vs. MAZIC Duro) within each thickness level. Because each subgroup consisted of independent specimens and only two groups were compared at a time, the independent t-test was the most appropriate method. Two‑way ANOVA was used to evaluate the combined and interaction effects of the two independent variables: (1) material type and (2) thickness, on each outcome (microhardness and fracture toughness). This test allowed us to determine not only the main effects of each factor but also whether a significant interaction existed between material and thickness an important aspect of the study design. All p-values were two-tailed, and statistical significance was set at p < 0.05.

## Results

### Hardness

#### Effect of different materials on thickness: subgroup analysis

**Fig. 2:**
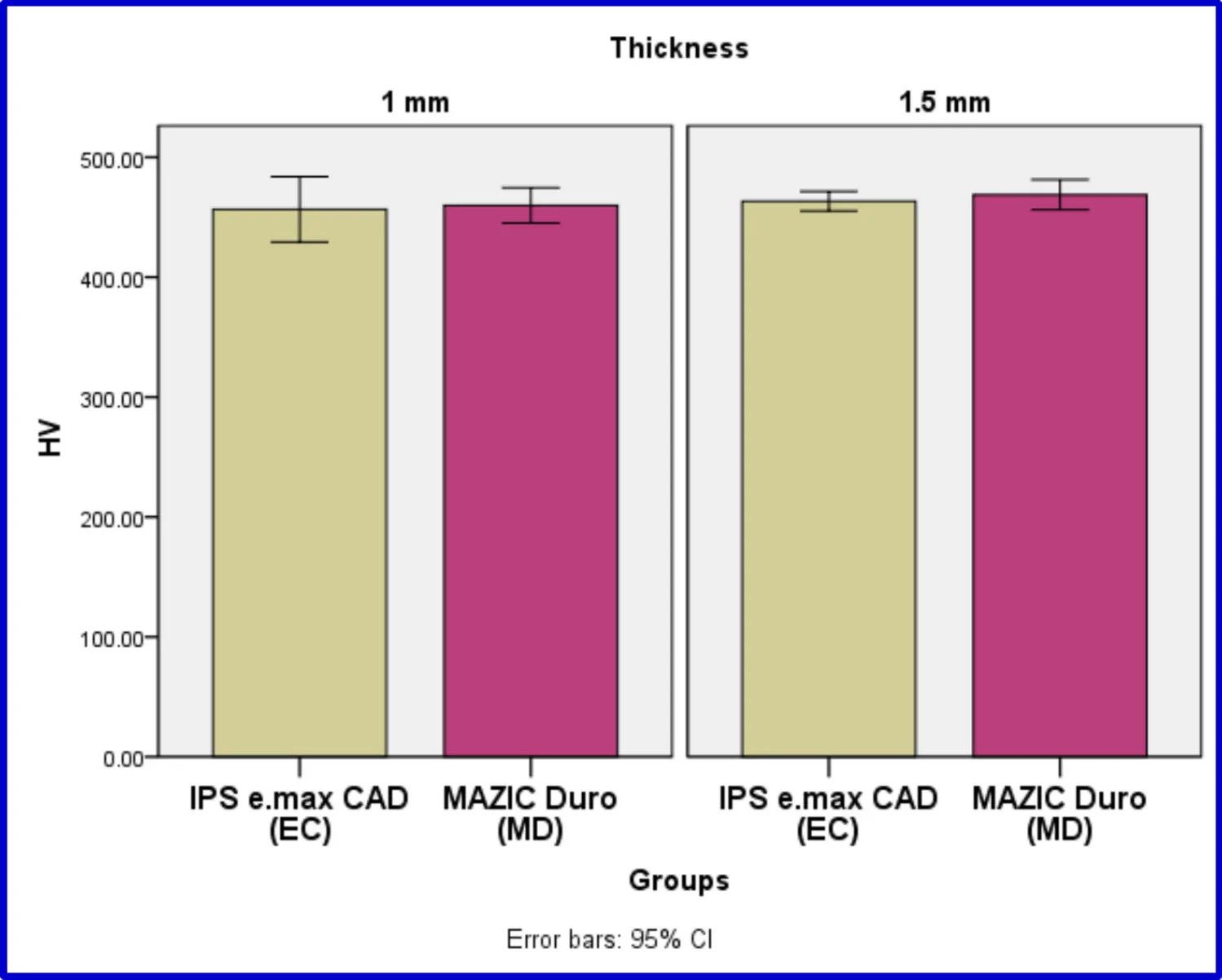
Bar chart illustrates mean Hardness (HV) of both materials and thicknesses

Across both **thicknesses (1 mm and 1.5 mm), MAZIC Duro (MD)** exhibited slightly higher mean hardness values compared to **IPS e.max CAD (EC);** however, these differences were not statistically significant. At 1 mm, MD recorded 459.836 ± 20.549 HV versus 456.596 ± 38.102 HV for EC (p = 0.816), and at 1.5 mm, MD recorded 468.818 ± 17.574 HV versus 463.346 ± 11.399 HV for EC (p = 0.420), as shown in Table 1 and Fig. 2.

**Table 1:** Descriptive statistics of hardness (HV) and comparison between groups (independent t test)

Thickness	Groups	Mean	Std. Dev	t test	P value	Thickness	Mean	Std. Dev	t test	P value
1 mm	IPS e.max CAD (EC)	456.596	38.102	−0.237	0.816 ns	1 mm	456.596	38.102	−0.54	0.603 ns
	MAZIC Duro (MD)	459.836	20.549			1.5 mm	463.346	11.399		
1.5 mm	PS e.max CAD (EC)	463.346	11.399	−0.826	0.420 ns	1 mm	459.836	20.549	−1.05	0.307 ns
	MAZIC Duro (MD)	468.818	17.574			1.5 mm	468.818	17.574		

Significance level p ≤ 0.05, ns = non-significant

Across both materials, increasing thickness from 1 mm to 1.5 mm resulted in slightly higher mean hardness values, although the differences were not statistically significant. For **IPS e.max CAD (EC),** the hardness increased from 456.596 ± 38.102 HV at 1 mm to 463.346 ± 11.399 HV at 1.5 mm (p = 0.603), while for **MAZIC Duro (MD),** the corresponding values increased from 459.836 ± 20.549 HV to 468.818 ± 17.574 HV (p = 0.307), as shown in Table 1 and Fig. 2.

#### Interaction of groups and thickness variables

**Fig. 3:**
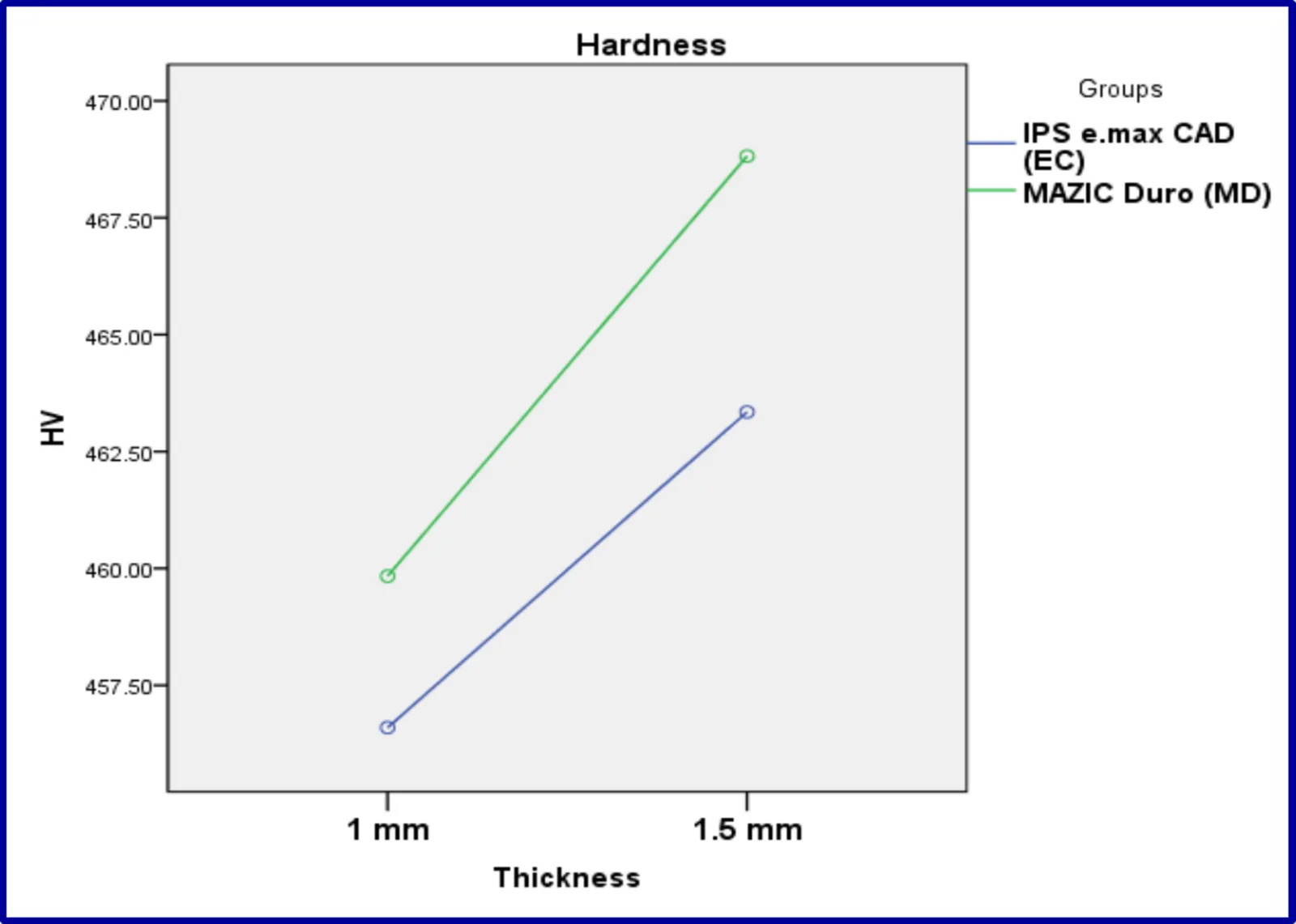
Line chart illustrating mean hardness (HV) of both materials and thicknesses

Two-way ANOVA indicated no statistically significant effect for the group factor (p = 0.570), thickness factor (p = 0.308), or their interaction (p = 0.884). Table 2 and Fig. 3.

**Table 2:** Results of two ways ANOVA test for the effect of groups and thickness variables on hardness (HV) values:

Source	Type III Sum of Squares	df	Mean Square	F value	P value	Partial Eta Squared	Observed Power
Groups	189.739	1	189.739	0.328	0.570 ns	0.009	0.086
Thickness	618.817	1	618.817	1.070	0.308 ns	0.029	0.172
Groups * Thickness	12.458	1	12.458	0.022	0.884 ns	0.001	0.052

Significance level p ≤ 0.05, ns = non-significant

### Fracture toughness

#### Effect of different materials on thickness: subgroup analysis

**Fig. 4:**
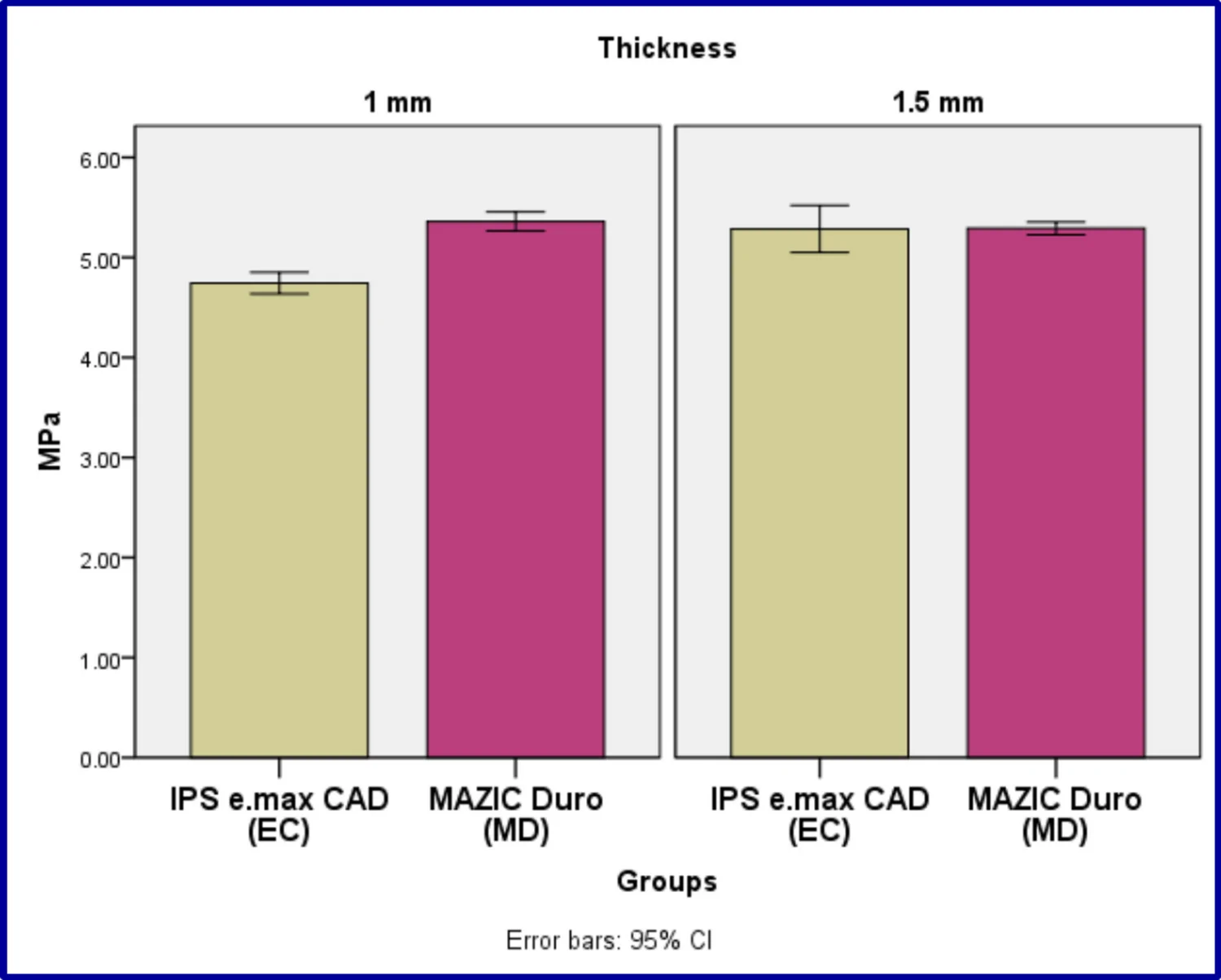
Bar chart illustrating mean fracture toughness (MPa.m^1/2^) of both materials and thicknesses

- **In subgroup (1mm):** MAZIC Duro (MD) recorded a highly statistically significant higher mean value of fracture toughness (5.360 ± 0.133 MPa.m^1/2^) compared to IPS e.max CAD (EC) (4.744 ± 0.151 MPa.m^1/2^), (p > 0.001). Table 3 and Fig. 4.
- **In subgroup (1.5 mm):** MAZIC Duro (MD) recorded a not statistically significant higher mean value of fracture toughness (5.291 ± 0.089 MPa.m^1/2^) compared to IPS e.max CAD (EC) (5.285 ± 0.330 MPa.m^1/2^), (p = 0.956). Table 3 and Fig. 4.

**Table 3:** Descriptive statistics of fracture toughness (MPa.m^1/2^) and comparison between groups (independent t test):

Thickness	Groups	Mean	Std. Dev	Difference				t test	P value
				Mean	Std. Dev	C.I. lower	C.I. upper		
1 mm	IPS e.max CAD (EC)	4.744	0.151	−0.616	0.064	−0.750	−0.482	−9.69	0.000**
	MAZIC Duro (MD)	5.360	0.133						
1.5 mm	IPS e.max CAD (EC)	5.285	0.330	−0.006	0.108	−0.246	0.234	−0.056	0.956 ns
	MAZIC Duro (MD)	5.291	0.089						

Significance level p ≤ 0.05, ** highly significant, ns = non-significant

#### Effect of thickness: subgroup comparison across material groups

- **IPS e.max CAD (EC) group:** 1.5 mm recorded a highly statistically significant higher mean value of fracture toughness (5.285 ± 0.330 MPa) compared to 1 mm (4.744 ± 0.151 MPa.m^1/2^) (p > 0.001). Table 4 and Fig. 4.
- **MAZIC Duro (MD) group:** 1 mm recorded a not statistically significant higher mean value of fracture toughness (5.360 ± 0.133 MPa) compared to 1.5 mm (5.291 ± 0.089 MPa.m^1/2^) (p = 0.190). Table 4 and Fig. 4.

**Table 4:** Comparison between thickness 1 mm and 1.5mm regarding fracture toughness (MPa.m^1/2^) (Independent t test)

Groups	Thickness	Mean	Std. Dev	Difference				t test	P value
				Mean	Std. Dev	C.I. lower	C.I. upper		
IPS e.max CAD (EC)	1 mm	4.744	0.151	−0.541	0.115	−0.790	−0.292	−4.71	0.000**
	1.5 mm	5.285	0.330						
MAZIC Duro (MD)	1 mm	5.360	0.133	0.069	0.051	−0.037	0.176	1.36	0.190 ns
	1.5 mm	5.291	0.089						

Significance level p ≤ 0.05, ** highly significant, ns = non-significant

#### Interaction of groups and thickness variables

**Fig. 5:**
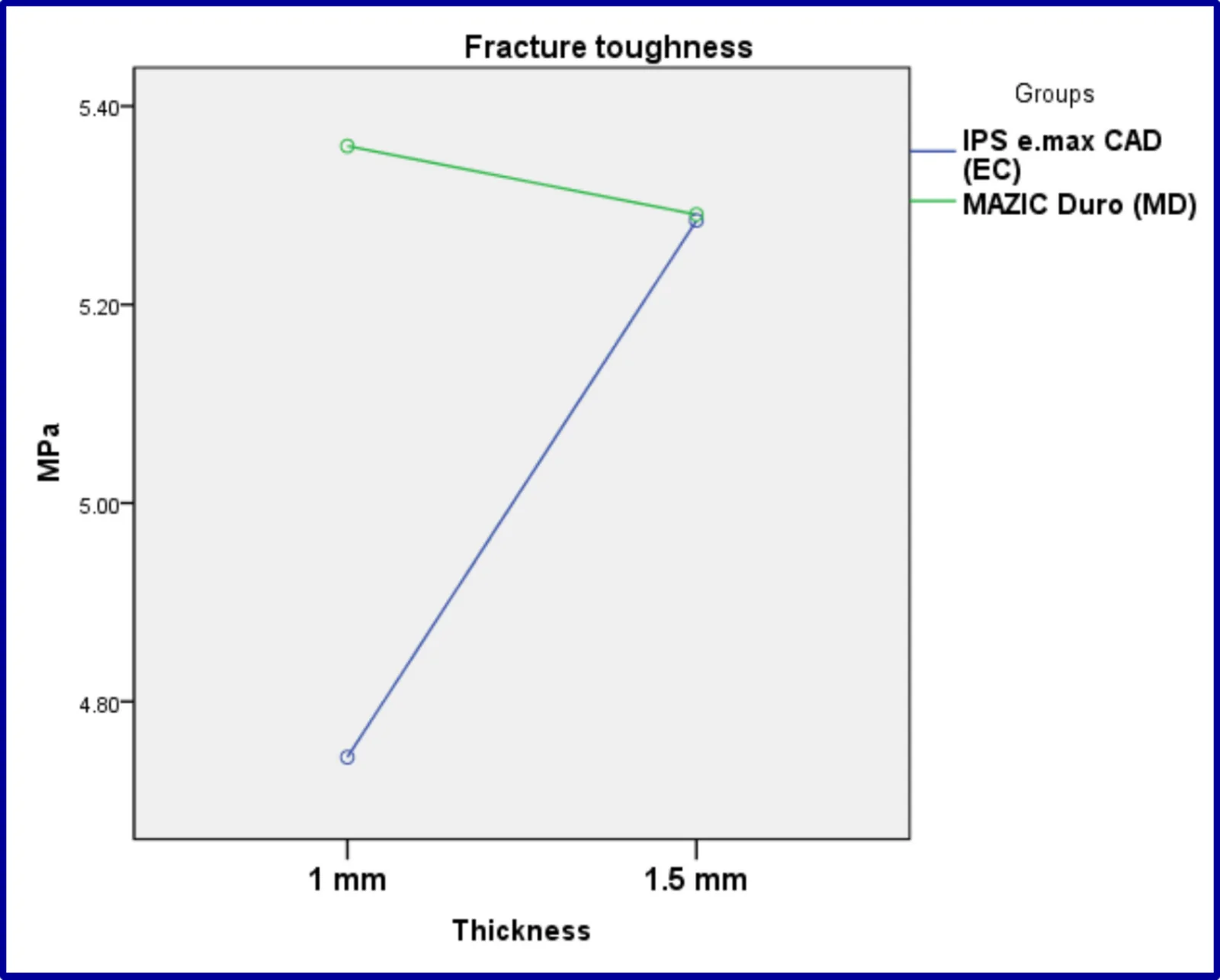
Line chart illustrates fracture toughness (MPa.m^1/2^) f both materials and thicknesses

Two ways ANOVA revealed a highly statistically significant effect for the groups’ variable (p > 0.001), thickness variable (p = 0.001) or their interaction (p > 0.001). Table 5 and Fig. 5.

**Table 5:** Results of two ways ANOVA test for the effect of groups and thickness variables on fracture toughness (MPa) values

Source	Type III Sum of Squares	df	Mean Square	F	P value	Partial Eta Squared	Observed Power
Groups	0.967	1	0.967	24.583	0.000**	0.406	0.998
Thickness	0.556	1	0.556	14.143	0.001**	0.282	0.955
Groups * Thickness	0.930	1	0.930	23.634	0.000**	0.396	0.997

Significance level p ≤ 0.05, **highly significant

## Discussion

This study examined the effect of varying thickness on the mechanical behavior of the material of two CAD/CAM blocks with different chemical compositions (IPS e.max CAD and MAZIC Duro) in terms of hardness and fracture toughness. The null hypothesis was rejected for hardness outcomes, whereas it was partially accepted for fracture toughness results.

In restorative dentistry, hardness represents a fundamental mechanical property that reflects a material’s ability to withstand surface indentation, finishing and polishing procedures quality, which are critical for clinical performance and esthetic outcome (Mörmann WH et al. 2013; Lawson NC et al. 2016). Microhardness was assessed by indenting the polished surface with a pyramidal diamond tip under controlled load, and hardness values were derived from the measured indentation dimensions (Asaad R et al. 2021).

Hardness results revealed no significant difference within materials at different thicknesses (1, 1.5 mm). To accurately interpret the hardness data of ceramic materials, several factors must be taken into account, including the type and distribution of crystalline phases, grain size, the influence of grain boundaries, and the presence of surface porosity (Egilmez F et al. 2018; Wang L et al. 2012).

These microstructural characteristics can significantly affect the material’s mechanical response and overall performance. MAZIC Duro achieves no significant difference compared to IPS e.max CAD in vickers hardness test due to its substantial concentration of ceramic fillers, including zirconia and silicate nanoparticles (Silva EA et al. 2023). This study is limited by the absence of aging, thermocycling, and mechanical fatigue testing, which may affect the long-term applicability and durability of the findings.

Albero A et al. (2015) reported that resin ceramics exhibit significantly lower Vickers hardness compared to glass ceramics, which contrasts with our findings. This variation may be explained by differences in testing protocols. Furthermore, the lower hardness and fracture toughness observed in ceramic–resin composites compared to glass ceramics could be attributed to their reduced inorganic content.

Restorations exhibit a tendency to fracture, with ceramic materials prone to breakage and resin-based materials increasing the risk of damage to the dental substrate, raising concerns about fracture resistance. Moreover, ceramic restorations are particularly vulnerable to machining-induced defects, especially in areas of minimal thickness such as restoration margins (Zhang Y et al. 2016).

Fracture toughness serves as a reliable indicator of a brittle material’s inherent resistance to crack initiation and propagation. It also reflects the material’s capacity to absorb strain energy generated by external forces, which is critical for maintaining structural integrity under stress (Rai P.et al. 2020: Marinis Aet al. 2011).

The indentation method was employed in this study to determine the fracture toughness of the two tested dental ceramics because it offers simplicity, reliability, and requires only small-sized specimens. Moreover, the cracks generated during testing closely mimic those typically encountered in clinical situations (Quinn JBet al 2010; Johnson Caet al. 1983).

In subgroup (1mm): MAZIC Duro (MD) recorded a highly statistically significant higher mean value of fracture toughness (5.360 ± 0.133 MPa) compared to IPS e.max CAD (EC) (4.744 ± 0.151 MPa), (p = 0.000). Table 4 and Fig. 4. In subgroup (1.5 mm): MAZIC Duro (MD) recorded a not statistically significant higher mean value of fracture toughness (5.291 ± 0.089 MPa) compared to IPS e.max CAD (EC) (5.285 ± 0.330 MPa), (p = 0.956). Table 3 and Fig. 4, this may be attributed to the chemical composition of IPS e.max CAD differing from that of MAZIC Duro, leading to increased residual tensile stresses within the glassy matrix. Consequently, these stresses compromise the material’s resistance to deformation and consequently result in a reduction in fracture toughness of the glass–ceramics (Li D et al. 2016). At reduced thickness (1 mm), stress concentration and limited bulk support amplify the influence of microstructural differences, favoring MAZIC Duro’s performance. Conversely, at 1.5 mm, the increased cross-sectional area provides greater structural support and distributes stresses more evenly, minimizing the impact of compositional differences and leading to comparable fracture toughness between the two materials.

Also, IPS e.max CAD demonstrates higher brittleness compared to ceramic–polymer composites, indicating that the resin matrix in MAZIC Duro may introduce a toughening mechanism within its microstructure (Zenaida S et al. 2021). Fracture toughness is improved by the high-density resin matrix, which provides some elasticity, and by the incorporation of nanosized ceramic fillers that contribute additional strength to the material. A previous study suggested that organic components (organic phase) help dissipate chewing forces, thereby improving the material’s flexural strength. During machining, a significant number of surface cracks and flaws are formed (Gracis S et al. 2015; Quinn G. et al. 2012).

A study comparing CAD/CAM blocks (Vita Enamic, Lava Ultimate, and MAZIC Duro) reported that MAZIC Duro and Lava Ultimate exhibited significantly higher biaxial flexural strength than Vita Enamic, indicating better resistance to fracture under load. Additionally, these materials showed smoother surfaces and less wear on opposing teeth after simulated brushing and wear tests (Yin et al., 2019).

Research on fracture toughness (KIc) of CAD/CAM materials shows that lithium disilicate ceramics (IPS e.max CAD) have the highest fracture toughness values among ceramics, while resin-based and hybrid ceramics (including Vita Enamic, Lava Ultimate, and similar materials) fall into a lower but comparable range. Although MAZIC Duro was not explicitly listed in this fracture toughness study, its composition and performance suggest it aligns with other resin-ceramic hybrids, offering improved toughness compared to glass ceramics but less than lithium disilicate (Hampe R et al. 2019), but the results in the current study were different and disagree with those of other resin based and hybrid ceramics.

Finally, this research found that no significant interaction effect was observed between material type and thickness for microhardness, indicating that changes in thickness influenced both materials in a similar manner. In contrast, a significant interaction effect was found for fracture toughness, demonstrating that the impact of thickness differed between the two materials. Specifically, MAZIC Duro showed a notably higher fracture toughness at 1 mm, whereas IPS e.max CAD did not exhibit a similar enhancement at reduced thickness.

This finding supports minimally invasive restorative dentistry by demonstrating that reducing the thickness of IPS e.max CAD and MAZIC Duro from 1.5 mm to 1 mm does not compromise surface durability or wear resistance. Because microhardness remained stable across thicknesses, clinicians can confidently use thinner restorations in areas of limited occlusal space without increasing the risk of surface wear or degradation. This enables more conservative tooth preparation while maintaining functional longevity of the restoration.

The finding that MAZIC Duro demonstrates significantly higher fracture toughness at 1 mm compared to IPS e.max CAD indicates that MAZIC Duro may be better suited for thin restorations, such as veneers, partial‑coverage restorations, or cases with restricted occlusal space. This suggests that MAZIC Duro may allow for a lower safe minimum thickness without compromising structural stability, whereas lithium disilicate may require adherence to standard thickness recommendations to avoid fracture risk.

Clinically, this finding highlights the importance of considering material composition and thickness when selecting restorations to minimize fracture risk. Although these findings provide valuable insights, clinical follow-up studies on ceramic–resin composites remain lacking. Therefore, future in vivo and in vitro investigations are essential to determine their durability and long-term behavior.

## Limitations

Despite the significant findings of this study, some limitations should be acknowledged. First, the sample size of ten specimens per subgroup, although statistically justified by power analysis and aligned with standard mechanical testing protocols, remains relatively small. A larger sample size could provide a broader representation of the materials' behavior under various conditions. Furthermore, this study was conducted in a controlled laboratory environment; therefore, future clinical studies are necessary to evaluate the long-term performance and fatigue resistance of these materials under complex intraoral conditions.

## Conclusions

MAZIC Duro demonstrated slightly higher hardness values than IPS e.max CAD at both 1 mm and 1.5 mm thicknesses; however, these differences were not statistically significant (p = 0.816 and p = 0.420, respectively). For fracture toughness, MAZIC Duro showed a significantly higher value at 1 mm thickness (p < 0.001), while at 1.5 mm thickness the two materials performed similarly, with no significant difference observed (p = 0.956). These findings suggest that material type and thickness influence mechanical performance, with MAZIC Duro presenting a distinct advantage at reduced thickness.

## Supplementary Information

Below is the link to the electronic supplementary material.

Supplementary file1 (GIF 29 KB)

Supplementary file2 (GIF 19 KB)

Supplementary file3 (GIF 26 KB)

Supplementary file4 (GIF 20 KB)

## Data Availability

No datasets were generated or analysed during the current study.

## Abbreviations

*MD*: MAZIC Duro
*EC*: IPS e.max CAD
*CAD/CAM*: Computer added design/Computer added manufacture
*HV*: Vickers hardness
*K*_*IC*_: Fracture toughness

## Declarations

Ethical approval for this study was obtained from the Research Ethics Committee of the Faculty of Dental Medicine for Girls et al.-Azhar University under code (REC-PD-25–28).
