# Correction: Surface and fracture properties of lithium disilicate and resin-Matrix CAD/CAM ceramics

**DOI:** 10.1007/s44445-026-00225-0

**Published:** 2026-09-03

**Authors:** Ayat G. Montaser, Sara Tamimi, Shimaa A. El Saeed, Ahmed D. Abogabal, Ahmed Y. Allam, Suad M. Hassan, Sally N. El Adawy, Amany H. Elzoka

**Affiliations:** 1 Crowns and Bridges Department, Faculty of Dental Medicine for Girls, Al-Azhar University, Cairo, Egypt; 2 Dental Biomaterials Department, Faculty of Dental Medicine for Girls, Al-Azhar University, Cairo, Egypt; 3 Department of Dental Biomaterials, Faculty of Dental Medicine (Boys), Al-Azhar University, Assiut, Egypt; 4 Crowns and Bridges Department, Faculty of Dental Medicine (Boys), Assiut, Al-Azhar University, Egypt., Assiut, Egypt; 5 Department of Dental Basic Science, Dental Biomaterials, Faculty of Dentistry, Petra University, Amman, Jordan

**Correction: The Saudi Dental Journal (2026) 38:54**

**https://doi.org/10.1007/s44445-026-00145-z**

The original publication of this paper has been updated as the following author was inadvertently omitted from the author list:

Amany H. Elzoka

Lecturer of Dental Biomaterials Department, Faculty of Dental Medicine for Girls, Al Azhar University

