# Supplementary figures and images for: Surface and fracture properties of lithium disilicate and resin-Matrix CAD/CAM ceramics
Source: Saudi Dent J. 2026 Apr 25;38(5):54. doi: 10.1007/s44445-026-00145-z (PMC13110258; doi:10.1007/s44445-026-00145-z)

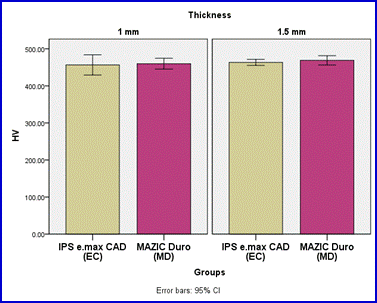

Supplement: Supplementary file 1 — Supplementary file1 (GIF 29 KB) [file 44445_2026_145_MOESM1_ESM.gif]

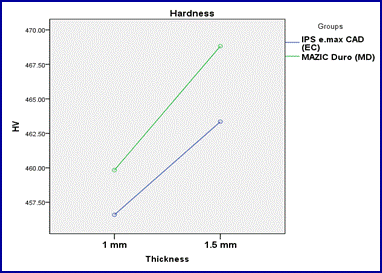

Supplement: Supplementary file 2 — Supplementary file2 (GIF 19 KB) [file 44445_2026_145_MOESM2_ESM.gif]

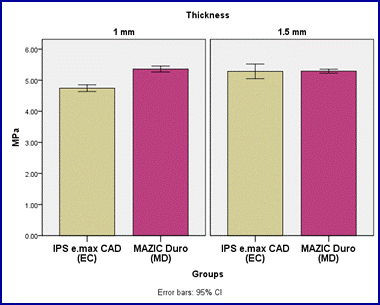

Supplement: Supplementary file 3 — Supplementary file3 (GIF 26 KB) [file 44445_2026_145_MOESM3_ESM.gif]

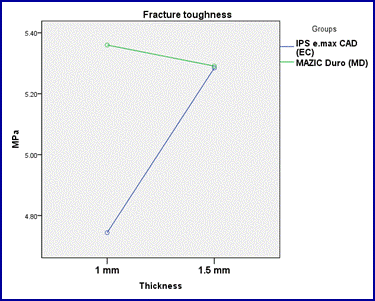

Supplement: Supplementary file 4 — Supplementary file4 (GIF 20 KB) [file 44445_2026_145_MOESM4_ESM.gif]
